# Assessment of a Novel Real-Time Bio-Liquor Circulation System for Manure Management and Mitigation of Odor Potential in Swine Farming

**DOI:** 10.3390/ani13243849

**Published:** 2023-12-14

**Authors:** Seungsoo Kim, Soomin Shim, Seunggun Won, Changsix Ra

**Affiliations:** 1 Department of Animal Industry Convergence, College of Animal Life Sciences, Kangwon National University, Chuncheon 24341, Republic of Korea; seungsoo89@kangwon.ac.kr (S.K.); smshim@kangwon.ac.kr (S.S.); 2 Department of Animal Resources, College of Life and Environmental Science, Daegu University, Gyeongsan 38453, Republic of Korea; swon@daegu.ac.kr

**Keywords:** swine farms, circulation system, greenhouse gas emission, bioreactor, manure treatment, oxidation reduction potential

## Abstract

**Simple Summary:**

The rise of malodor complaints from residents around swine farms and greenhouse gas (GHG) emissions from manure have become a global concern. Although various technologies have been developed to reduce gas emissions from swine farms, only a few have been successfully applied. In Korea, a bio-liquor circulation system (BCS) that controls odor by diluting slurry pit manure with biologically treated manure (bio-liquor) has been proposed as an odor control technology. However, the successful implementation of BCS has been hindered due to the lack of suitable bioreactor control strategies. Therefore, to effectively use BCS, it is crucial to develop an optimized control technology for bioreactors and system operations. Hence, this study aimed to evaluate the effectiveness of a novel real-time BCS controlled by tracking the time profiles of the oxidation reduction potential (ORP) and pH (mV) within two bioreactors in an experimental swine farm set up to improve manure treatment efficiency and reduce malodor at farm scale. The study’s results showed that the real-time BCS successfully controlled and optimized the bioreactor and the entire process, effectively reducing the concentrations of nitrogen and organic matter in the swine manure in the slurry pit, and subsequently the malodor.

**Abstract:**

Recently, circulating biologically treated manure in slurry pits has been used as an odor reduction technology, but few successful results have been reported, due to the lack of proper control strategies for bioreactors. This study was conducted to investigate the performance of the developed real-time controlled bio-liquor circulation system (BCS) at farm scale. The BCS was operated sequentially as per swine manure inflow (anoxic, aerobic, and settling) circulation to the slurry pit. Each operational phase was self-adjusted in real-time using a novel algorithm for detecting the control point on the oxidation reduction potential (ORP) and pH (mV)–time profiles, the nitrogen break point (NBP), and the nitrate knee point (NKP) in the aerobic and anoxic phases, respectively. The NH_4_-N in the slurry manure was thoroughly removed (100%) in the bioreactor, optimizing the duration of each operational phase by accurately detecting real-time control points. The newly developed real-time BCS decreased the nitrogen and organic matter in the slurry pit by >70%, and the potential ammonia and methane emissions by 75% and 95%, respectively. This study highlights that improved BCS that utilizes ORP tracking and pH (mV)–time profiles can effectively optimize BCS operation, and thereby reduce malodor and GHG emissions from swine farms.

## 1. Introduction

Globally, the livestock industry is widely known to cause various environmental concerns, and for complaints of unpleasant odors emanating from intensive livestock farms [1,2,3,4,5]. As the societal demand for a pleasant living environment increases, advances in the livestock industry are hindered by its odors [6,7,8]. However, controlling odors from livestock farms is challenging due to the complexity in structural problems that restrict the collection and treatment of odorous air and its associated high operating cost. It is estimated that an annual cost of USD 480–560 million was required to control malodors resulting from the Korean livestock industry [9]. 

Various odor reduction technologies using physical, chemical, and biological principles have been developed for the livestock industry [10,11]. Existing odor reduction technologies such as biofiltration, wet scrubbing, oil spraying, and UV light are excellently effective in removing specific odor compounds. However, odors from livestock farms contain a diverse range of complex gases [12,13]. Due to this complexity, these odor control technologies have very low or no treatment efficiency in terms of livestock-related odor removal, thereby making odor control in livestock facilities highly challenging [14]. Therefore, a fundamental control solution is required to effectively manage odors in the livestock industry.

In odor management, the control of odor sources such as manure is the most obvious target with the highest priority [15]. Odor from livestock manure is caused due to the presence of incomplete anaerobic biodegradation of undigested nutrients, endogenous secretions, and the intermediate and final products of the intestinal microflora [16,17,18]. Organic matter in swine manure generates four groups of volatile odorous gases such as VFAs (volatile fatty acids), aromatic compounds (indoles and phenols), nitrogen compounds, and sulfur compounds through hydrolysis and microbial fermentation. VFAs are produced by fermentation of carbohydrates and amino acids; further, some amino acids (tyrosine, phenylalanine, tryptophan) contribute to the generation of aromatic compounds. Nitrogen compounds such as ammonia and volatile amines are produced by decarboxylation of amino acids, and specific amino acids (methionine and cysteine) are precursors which generate volatile sulfur compounds such as mercaptans and hydrogen sulfide. Furthermore, urea, which is abundantly contained in urine undergoes hydrolysis to NH_3_ through the action of the enzyme urease [15]. Schiffman et al. highlighted that since 331 different types of volatile organic compounds are complexly emitted from swine facilities, not only odor issues, but also medical consideration of the health of animals and people, are necessary [19]. Furthermore, because CO_2_ and CH_4_ are emitted in the final process of fermentation, the swine industry must strive to reduce greenhouse gases (GHG) as well as odor. Therefore, it is important to suppress anaerobic microorganism activity in manure to prevent odor release from livestock manure. Subsequently, various methods have been suggested for odor source control, including dietary control, microbial additives, acidification, and aeration control. However, each method has limitations. For example, dietary control is an odor source control method that decreases indigestible nutrients in swine manure by increasing the digestibility of feed in pigs; however, the odor reduction effect is not high, and its underlying mechanism remains unexamined [20,21]. Acidification suppresses ammonia (NH_3_) emissions by increasing the ratio of ammonium (NH_4_^+^) in swine manure, while hydrogen sulfide (H_2_S) emissions increase [22,23,24]. According to Zhang and Zhu, intermittent aeration can reduce the emissions of volatile fatty acids (VFAs) by approximately 45–95%; however, a side effect of increased NH_3_ emissions and overflowing foam was observed [25]. Several researchers have observed that the use of commercial microbial additives in slurry pits does not show consistent odor reduction effects, and thereby suggesting that not all microbial products are effective in reducing odors [26,27]. Overall, although the methods of odor source control apparently reduce odor, its underlying mechanism is yet to be understood, and each method has adverse effects. Therefore, these technologies may not be suitable realistic solutions for controlling high-intensity odors on swine farms.

Recently, in Korea, the management of slurry pits mixed with the supernatant of biologically treated manure (bio-liquor) has been highlighted as a source control technique, and is called a bio-liquor circulation system (BCS), pit recharge system, or liquid manure circulation system [28,29,30,31,32,33,34]. Despite their different names, the common aim of these systems is to lower the concentration of the odor source in the slurry pit by dilution with a bio-liquor. Notably, for the effective reduction of odor sources in swine manure using BCS, proper treatment of slurry manure in a bioreactor and the circulation of a sufficient amount of well-treated bio-liquor for dilution is essential. However, the optimal state of the bioreactor is difficult to maintain, owing to the fluctuating microbial activity in the bioreactor and the changing characteristics of the slurry manure. Accordingly, Wi et al. [31] argued that implementing a highly automated system for operating a BCS is unlikely. 

Therefore, this study aimed to validate the farm-scale applicability of a novel real-time control strategy of a BCS that utilizes identifying unique points on oxidation reduction potential (ORP) and pH–time profiles to evaluate the control stability of the implemented system and its effects on potential odorous and greenhouse gas emissions from slurry pits at farm scale. This method has only been previously developed and demonstrated at pilot scale [28,29,30].

## 2. Materials and Methods

### 2.1. The Bio-Liquor Circulation Process

**Figure 1:**
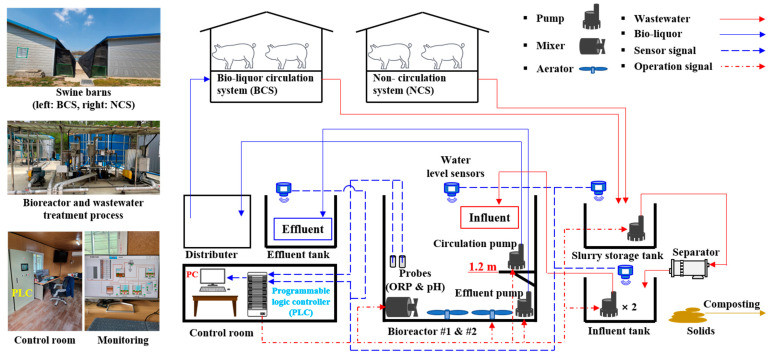
Bio-liquor circulation process in the experimental swine farm.

The experiment was conducted for 12 weeks starting on 7 January 2022, at a swine farm in Eumseong-gun, Chungcheongbuk-do, Republic of Korea. The experimental farm was equipped with two swine barns, two bioreactors, a slurry storage tank, an influent tank, an effluent tank, a solid–liquid separator, and a bio-liquor distributor. Detailed information regarding the process structure and scale are presented in Figure 1 and Table 1, respectively. 

One swine barn was categorized as a non-circulation system (NCS) and the other as a BCS, depending on the presence of bio-liquor circulation from the bioreactor to the slurry pit. There were 96 and 128 pigs in the NCS and BCS barns, respectively, and the pig density remained the same at 0.29 head/m^2^ due to the difference in areas. Considering swine manure production to be 5.1 L/head/day, as estimated by the Ministry of Environment in Korea, the manure production in swine barns was calculated to be 489.6 L/day and 652.8 L/day, respectively. The total volumes of the NCS slurry pit (NCS-SP) and BCS slurry pit (BCS-SP) in each swine barn were 212.9 m^3^ and 286.2 m^3^, respectively, and the working volume was adjusted to 61.5% of the total volume in both slurry pits by limiting the effective height to 0.4 m. Consequently, the volume of each slurry pit per head was similar, with 2.28 m^3^/head in NCS and 2.24 m^3^/head in BCS.

The swine manure from both NCS-SP and BCS-SP was moved by gravity to the slurry storage tank, where the solid and liquid fractions were separated. The solid fraction was transferred to a composting lot, whereas the liquid fraction was stored in the influent tank. Wastewater in the influent tank was fed into the bioreactor, and the supernatant from the bioreactor after biological treatment was circulated to the slurry pit through a distributor. The distributor was constructed to evenly distribute the bio-liquor to the three compartments of the BCS-SP. 

#### 2.1.1. Slurry Pit Remodeling

Both the NCS and BCS swine barns were divided into three categories: fattening room 1 (FR1), fattening room 2 (FR2), and piglet room (PR). Pig breeding and manure storage in both the barns were conducted individually for each compartment. 

**Figure 2:**
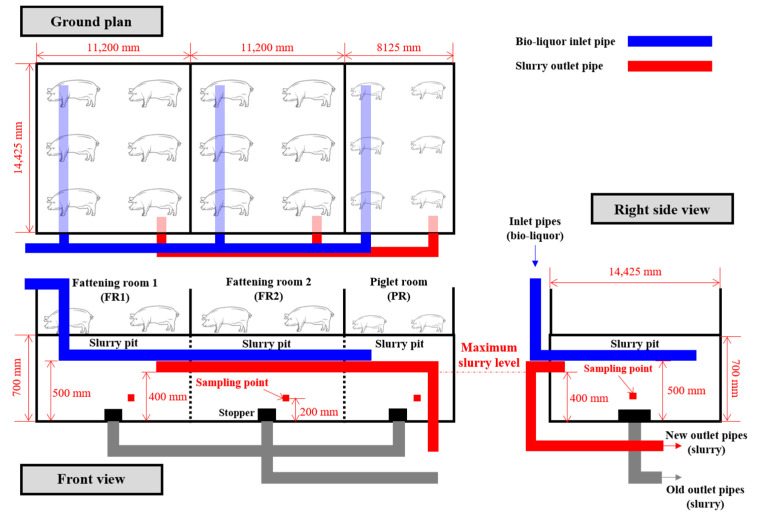
Structure of circulation pipelines in the swine barn.

Circulation pipes were installed in the BCS-SP to distribute the bio-liquor equally in each compartment (Figure 2). The BCS-SP was installed with additional inlet and outlet pipes made of poly-ethylene (∅ 50 mm) for the real-time circulation based on the preset algorithm. The inlet and outlet pipes for bio-liquor circulation were placed at heights of 500 and 400 mm from the bottom of the slurry pit, respectively. In addition, the inlet and outlet pipes were located on opposite sides of the slurry pit to avoid flux passing and ensure uniform circulation. When the water level was increased by the circulation of the bio-liquor, the slurry manure and bio-liquor mixture naturally overflowed into the slurry storage tank through the outlet pipe. In contrast, in NCS-SP, the slurry manure was discharged into the slurry storage tank by opening a stopper in each compartment when the slurry pit was full, as in conventional practice.

#### 2.1.2. Construction of the Bio-Liquor Distributor

**Figure 3:**
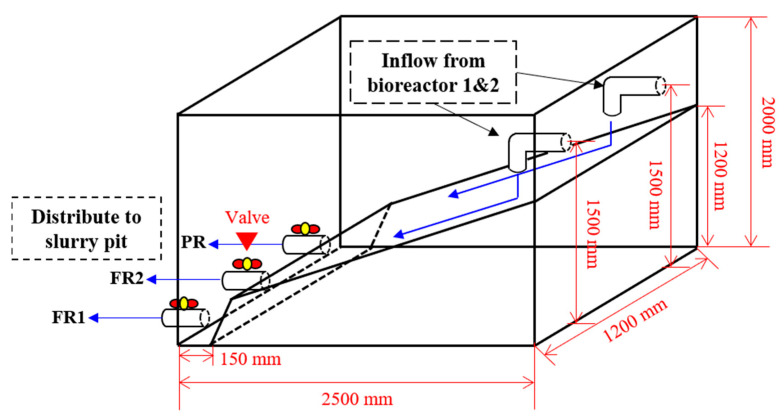
Schematic layout of the bio-liquor distributor.

As shown in Figure 3, the distributor had a total volume of 6 m^3^ and the influxes from bioreactors 1 and 2 sloped down to the front of the outlet, connecting to FR1, FR2, and PR. After the bio-liquor flowed from the bioreactor, it gathered in the lower part of the distributor, where the valve was located, and flowed into each compartment of the slurry pit through the pipes connected to FR1, FR2, and PR. Furthermore, polyvinyl chloride valves were installed on each circulation pipe connecting the BCS-SP and the distributor to control the inflow of the bio-liquor into each compartment.

#### 2.1.3. Construction of the Bioreactor and Bio-Liquor Circulation System

To construct a real-time-controlled BCS, several sensors and apparatus were installed, and the system was operated using an algorithm connected to it through a programmable logic controller (PLC, XBC-DR28U, LS Electric Co., Ltd., Seoul, Republic of Korea) located in the control room (Figure 1).

The two bioreactors, with an individual floor area of 16.7 m^2^ and an effective height of 2.4 m, were equipped with the actuators necessary for their operation. These included an ORP sensor (SOR-400G platinum ring Ag/AgCl, Samsan Korea, Gyeonggi-do, Republic of Korea), a pH sensor (SPH-200G glass body Ag/AgCl, Samsan Korea, Gyeonggi-do, Republic of Korea), a water level sensor (FMR52-ICACCDBPAGK, Endress + Hauser AG, Kägenstrasse, Switzerland), and an aerator, mixer, and pump. The ORP and pH probes were installed at a height of 1.2 m, corresponding to the middle water level in the bioreactor. They were placed in a location to avoid direct contact with air. The aerator supplied air at a rate of 0.1 m^3^ air/min/m^3^ reactor through 18 air diffusors installed at the bottom of the bioreactor. To prevent the formation of a dead zone inside the bioreactor and ensure uniform bioreaction, a mixer was installed at one corner of the bottom. Two pumps were installed at different locations in the bioreactor to transfer the bio-liquor to the slurry pit after treatment, and the bio-liquor equivalent to the amount of manure excreted was pumped into the effluent tank. The effluent pump was positioned at the bottom to transfer the bio-liquor containing the settled activated sludge to the effluent tank. The other pump was installed 1.2 m above the bottom of the bioreactor to circulate the supernatant into the slurry pit after settling (the bioreactor operation method is explained in Section 2.2).

Water level sensors were installed on top of the bioreactor, slurry storage, influent, and effluent tanks to determine the operation times of the pumps for wastewater inflow and treated water discharge. The types of pumps in the system and their operation times were determined based on measurements from the water level sensors, ensuring that overflow was prevented in each tank.

### 2.2. Construction of the Bioreactor and Bio-Liquor Circulation System

#### 2.2.1. Monitoring System

For real-time control of the BCS, the time profiles of the ORP and pH in the bioreactor and the water level sensors in each tank were continuously monitored. The values measured from each sensor were transmitted to the PLC as electrical signals ranging from 4 to 24 mA and visualized using a human–machine interface (HMI, AutoeYe smart, AUTO C&I Co., Ltd., Seoul, Republic of Korea). The ORP and pH-mV values were taken at 1 s intervals, but the data recording was conducted every minute by averaging the values over a 60 s period. The water level sensor accurately detected the level changes in increments of 1 mm, and the received signal was monitored at 1 s intervals, which resulted in immediate control of the pumps. The PLC controlled the entire BCS, including the wastewater inflow, bioreactor operation, bio-liquor circulation, and effluent storage, following a designated algorithm that tracked the time profile of the ORP and pH (mV).

#### 2.2.2. Operational Algorithm for the Bio-Liquor Circulation System

**Figure 4:**
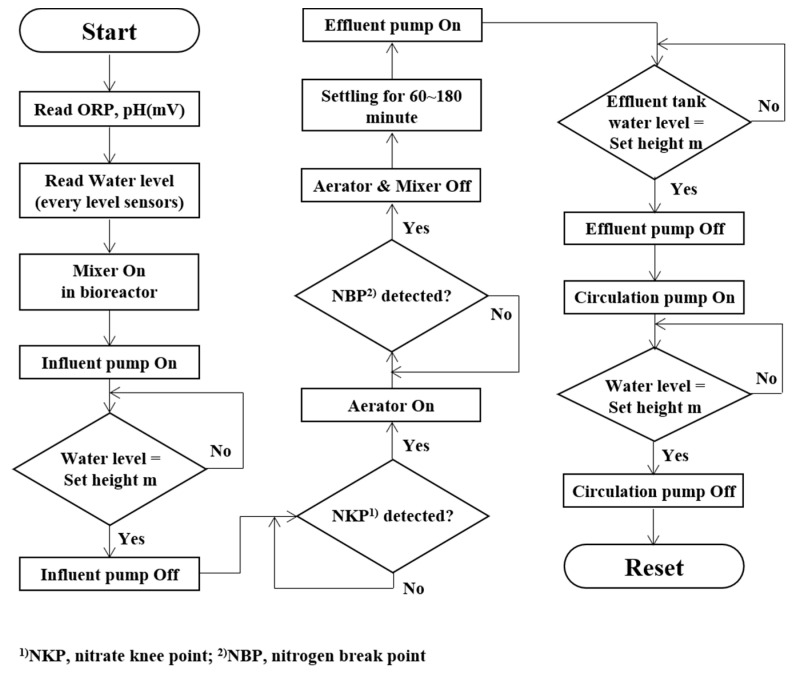
Real-time control algorithm of bio-liquor circulation system.

A real-time operational algorithm for the BCS is shown in Figure 4. The operational concept of the bioreactor is the circulation of the bio-liquor with a focus on nitrogen behavior. Further details on the real-time detection of the nitrogen breakpoint (NBP) and nitrate knee point (NKP) were obtained by monitoring the ORP and pH–time profiles from previous research [29,30,35]. The bioreactor was operated repeatedly in the following sequence: feeding, mixing, NKP detection, aeration, NBP detection, settling, and circulation. Water level signals determine the amount of influent feeding, circulation of the bio-liquor, and discharge of the effluent. The pumps were operated according to the algorithm and stopped once the target water level was reached, ensuring a balance between the inflow and outflow in the bioreactor.

#### 2.2.3. Operational Conditions

The operating condition of the BCS is listed in Table 2. Initially, the bioreactors were filled with a mixture of bio-liquor and activated sludge (mixed liquor). Both bioreactors were individually operated under the same operational conditions to treat 0.6 m^3^ of wastewater per cycle based on the algorithm. When the NBP was detected in the bioreactor, the PLC circulated the bio-liquor after settling for 1 h in the slurry pit at a rate of 0.6 m^3^/cycle. Consequently, 3.4 L/m^3^ of bio-liquor based on the slurry pit volume was circulated in each cycle into the BCS-SP from each bioreactor. The initial characteristics of the mixed liquor in the bioreactors are summarized in Table 3.

### 2.3. Temperature Control and Breeding Program in the Swine Barns

The experimental farm operated using a continuous rearing system (no breeding was carried out for piglet production, and new piglets were purchased and introduced from the market). In the rearing program, new piglets were introduced after finishing, and the pigs were brought to the market to ensure a continuous stocking cycle. The NCS and BCS barns were designed to accommodate 100 pigs and 140 pigs, respectively. Pigs in both pig barns were maintained at 50% for piglets (PR) and 50% for growing fattening pigs (FR1 and FR2). Accordingly, when pigs were taken to the market from FR1 and FR2, the pigs in PR were moved to FR1 and FR2, whereas new piglets were introduced into PR. Most of the experimental conditions were the same for the NCS and BCS swine barns, except for the circulation of the bio-liquor. Table 4 shows the temperature range and feed composition in the NCS and BCS swine barns. In FR1 and FR2 of both swine barns, the temperature in each compartment was regulated between 20 and 25 °C, while in PR, it was maintained at 25–30 °C. The ventilation fans and warming lamps in each room were controlled using an automated controller to maintain the desired temperature range. Additionally, NCS and BCS received the same feed tailored for their respective growth stages, with an unlimited supply of feed and water.

### 2.4. Sampling Procedure

In this study, approximately 1 L of each sample was collected from the slurry pit, slurry storage tank, influent tank, and distributor at intervals of 2 weeks. 

To compare the differences in swine manure characteristics, 300 mL of swine manure was collected from each compartment (FR1, FR2, and PR) of the NCS and BCS. After collection, the samples were mixed according to the swine barn type to obtain representative NCS and BCS samples. The sampling points are shown in Figure 2. Bio-liquor samples were collected directly from the distributor during circulation, whereas the samples from the slurry storage and influent tanks were collected after operating the mixer for at least 5 min.

From the samples collected at the farm site, 200 mL was immediately filtered through filter paper (Whatman No. 6) after centrifugation at 3000 rpm for 10 min. The filtered and original samples were refrigerated below 4 °C after being transported to the laboratory, and further analyses were conducted within the week.

### 2.5. Analytical Methods

Total solids (TS), total volatile solids (TVS), total suspended solids (TSS), total volatile suspended solids (TVSS), and total Kjeldahl nitrogen (TKN) were analyzed. TS was determined by measuring the weight of the remaining sample after drying at 105 °C for 24 h, while TVS was analyzed by measuring the weight of the sample after volatilization in a muffle furnace at 550 °C for 4 h. TSS was determined by measuring the weight of the filtered solids obtained through a glass fiber filter after drying at 105 °C for 24 h. Subsequently, the weight of the volatilized sample after burning in a muffle furnace at 550 °C for 4 h was analyzed to determine TVSS. TKN was analyzed using an automated water analyzer (QuickChem 8600, Lachat, Weston, CT, USA) after adding sulfuric acid to the sample and digesting it at 380 °C for over 4 h.

The filtered samples were used to analyze soluble total organic carbon (STOC), ammonium nitrogen (NH_4_-N), and nitrogen oxides (NO_X_-N). STOC was analyzed using an automated TOC analyzer (Torch, Teledyne Tekmar, Mason, OH, USA). NH_4_-N and NO_X_-N were analyzed using an automated water analyzer (QuickChem 8600, Lachat, Weston, CT, USA). Total nitrogen (TN) was determined as the sum of the NO_X_-N and TKN measurements obtained from the water analyzer. Although all analyses were performed in this study, considering that the collected 1 L sample may not represent the entire slurry pit accurately, the interpretation of data was carried out primarily based on those of the filtered samples, which may be less influenced by the solid fraction.

### 2.6. Calculation of the Methane and Ammonia Emission Potentials of Swine Manure

The CH_4_ and NH_3_ emissions from swine manure were calculated according to the guidelines of the Intergovernmental Panel on Climate Change (IPCC) and the method proposed by Emerson et al. (Table 5) [36,37]. Potential CH_4_ emissions per unit volume of swine manure were calculated using Equation (1).

(1) $${CH}_{4}\;production\;(kg\;{CH}_{4}/m^{3}\;manure)=VS\;(kg/m^{3}\;manure)\times B_{o}\;(m^{3}\;{CH}_{4}/kg\;VS)\times MCF\;(\%)\times 0.67\;(kg/m^{3})$$

where

VS: concentration of volatile solids in swine manure;

B_o_: maximum CH_4_ production capacity (m^3^/kg of VS) for manure produced by pigs;

MCF: CH_4_ conversion factors for each manure management system;

0.67: CH_4_ conversion factor of m^3^ to kg.

The IPCC Tier II CH_4_ emission factors and values used to estimate the potential CH_4_ emissions from swine manure in NCS-SP and BCS-SP are listed in Table 5. For MCF, the temperatures in the swine barn in the NCS and BCS were fixed at 18 °C to correct for differences in CH_4_ emissions due to temperature. For the B_o_ value, the emission standard for swine markets in Western Europe adopted by the Ministry of Environment of Korea was used [38].

**Table 5:** References and calculation factors for estimating the potential emissions of CH_4_ and NH_3._

Parameters	Reference	Used Factor	Used Value
CH_4_	IPCC (2006) [36]	Volatile solids (VS): analyzed value	VS (kg/m^3^)
		B_o_ ^1^: Market swine characteristics in Western Europe	0.45 (m^3^ CH_4_/kg VS)
		MCF ^2^: Slurry system, 18 °C	35 (%)
		CH_4_ conversion factor of m^3^ to kg	0.67 (kg/m^3^)
NH_3_	Emerson et al. (1975) [37]	pH	measured value
		Temperature	18 (°C)

^1^ B_o_, maximum CH_4_ producing capacity; ^2^ MCF, CH_4_ conversion factors by manure management system for each climate region.

Potential NH_3_ emissions were estimated using the ratio of NH_3_ to NH_4_^+^ in manure by temperature and pH. Similar to the calculation of CH_4_ emissions, the temperature was fixed at 18 °C to exclude the effect of temperature on the pK_a_ value. Emerson et al. suggested that the NH_3_ ratio can be determined using Equation (2) [37].

(2) $$Percent\;of\;{NH}_{3}\left({NH}_{3}/{NH}_{4}-N\right)=1/(10^{{PK}_{a}-pH}+1)$$

## 3. Results and Discussion

### 3.1. Real-Time Operation of the Bioreactors

**Figure 5:**
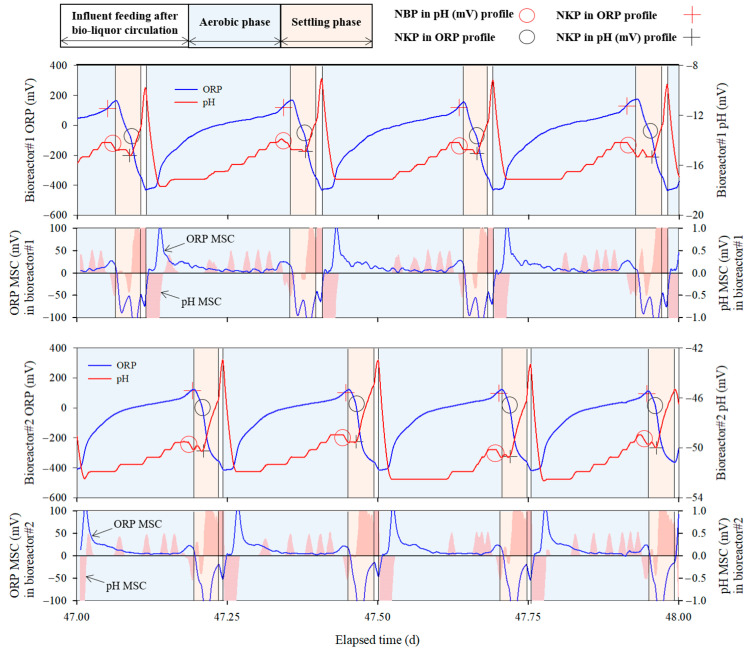
Representative ORP and pH (mV)–time profiles during operation in two bioreactors.

**Figure 6:**
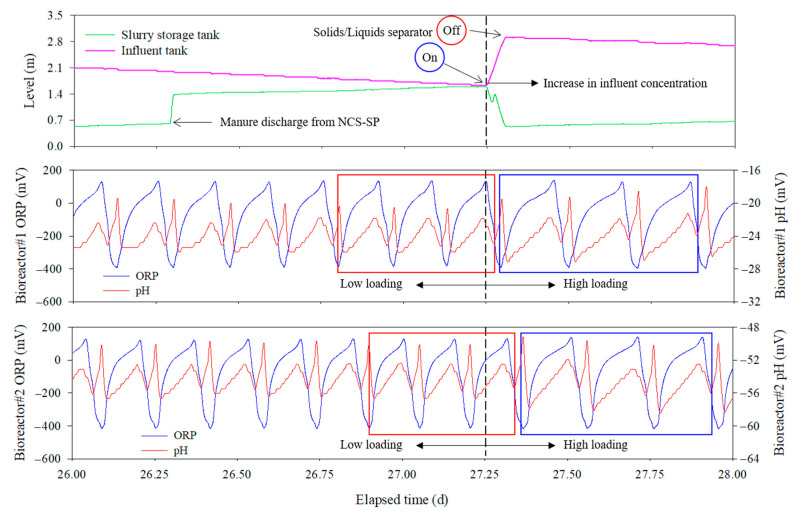
Changes in cycle duration of the real-time controlled bioreactor with the discharge of swine manure from non-circulating system slurry pit.

The performance and operational stability of real-time control using the ORP and pH (mV)–time profiles are shown in Figure 5 and Figure 6 and Table 6. During the aerobic phase, overall increases in ORP and pH (mV) were observed. ORP increased rapidly at the start of aeration and then increased slowly, whereas pH (mV) showed an overall steady increase. Towards the end of the aerobic phase, a specific pattern emerged wherein ORP increased rapidly again (+ symbol in Figure 5) and pH (mV) suddenly decreased (○ symbol in Figure 5). This specific pattern is known as the NBP, and indicates the completion of nitrification. Detailed information on the appearance of the NBP signals can be found in previous studies [28,34,38]. The PLC accurately detected NBP by identifying a clear reversal in the moving slope change (MSC) of pH (mV) transitioning from positive to negative. Following the algorithm, aeration was stopped, and the reactor was switched to the settling phase to prepare for the circulation of the bio-liquor. 

During the settling phase, NKP, which indicates the termination of denitrification, appeared in the ORP and pH (mV) time profiles. In this phase, ORP decreased rapidly below −300 mV (○ symbol in Figure 5) indicating NKP. However, the pH(mV) suddenly increased (+ symbol in Figure 5) at the point where NKP appeared in the ORP-time profile, indicating that NKP also appeared in the pH (mV)–time profile. Previous studies have confirmed that there are differences between ORP values under anoxic and anaerobic conditions [29,30,39,40,41]. When the ORP decreased below −300 mV, the bioreactor became anaerobic under anoxic conditions. Additionally, Tanwar et al. reported that the pH decreased under anaerobic conditions owing to fermentation [42]. 

In this study, as observed in the ORP and pH (mV) time profiles during the settling phase, denitrification was completed within an hour without external carbon source supplementation. These results contradict those of previous studies reporting that the long-term operation of real-time controlled BCS can result in a reduction in the organic carbon concentration in the manure within the slurry pit owing to the circulation of the bio-liquor, which could lead to a limited supply of the carbon sources necessary for denitrification, potentially resulting in prolonged denitrification [29,30]. However, in this study, complete denitrification was achieved without an external carbon source, which could be attributed to endogenous denitrification by microorganisms. The initial concentrations of mixed liquor suspended solids (MLSS) in this study were twice of that in the two previous studies, i.e., 20,760.0 ± 301.4 and 17,200.0 ± 202.1 mg/L for bioreactors 1 and 2, respectively (Table 3). Gao et al. found that introducing activated sludge into an anoxic tank without adding external carbon sources increased nitrogen removal efficiency through endogenous denitrification [43]. In this study, because bio-liquor, as the supernatant, was discharged by a circulation pump installed at 1.2 m height after settling, the MLSS concentration in the bioreactor was maintained at a high level.

Table 6 lists the MLSS, dissolved nitrogen, and STOC concentrations in the circulating bio-liquor in each bioreactor. The average MLSS concentration in the circulated bio-liquor from bioreactors 1 and 2 was 25,185.0 ± 3933.0 and 26,058.3 ± 5795.7 mg/L, respectively. Because the bio-liquor was circulated after settling for more than an hour, the concentration of MLSS within the bioreactor should be much higher than that of the circulated bio-liquor. When denitrification was complete during the settling phase of each cycle, the PLC promptly turned on the aerator after the influent feeding. 

As shown in Table 6, despite the NH_4_-N loading rates of 157.1 ± 43.0 and 192.9 ± 46.3 g/m^3^·d in bioreactors 1 and 2, respectively, the NH_4_-N concentration in the circulated bio-liquor remained consistent at 0.0 mg/L, demonstrating 100% removal efficiency throughout the experiment. The performance of the real-time controlled bioreactor was steadily maintained, and when the swine manure from the NCS-SP entered the influent tank, the load rate into the bioreactor increased. Figure 6 illustrates the manure flowing from the NCS-SP into the slurry storage and influent tanks, and the ability to self-regulate the processing time of the real-time controlled bioreactors in response to fluctuations in the influent concentration. As the bioreactor continued to treat the wastewater and circulate the bio-liquor, the level in the influent tank gradually decreased. When swine manure was discharged from the NCS-SP at approximately day 26.3, the water level of the slurry storage tank increased from 0.7 m to 1.4 m, and subsequently the water level in the influent tank rapidly increased at day 27.3 with the operation of the solid–liquid separator. 

A clear difference in cycle duration was observed before and after day 27.3, when high-concentration manure was loaded into the influent tank from NCS-SP, thereby increasing the concentration of the influent. Comparing the average duration of three cycles before and after 27.3 d (Figure 6), the average duration for each cycle before day 27.3 (red box in Figure 6) was 236.7 ± 3.5 and 223.7 ± 6.7 min in bioreactors 1 and 2, respectively; however, this was extended to 297.3 ± 2.5 and 283.7 ± 7.7 min/cycle after that day (blue box in Figure 6), respectively.

The results of this experiment demonstrate that wastewater treatment time varies with the loading rate of the bioreactor, which supports the argument of Wi et al. that automating BCS using conventional methods is impossible [31]. Conventional BCS are primarily operated using quantitative control methods that provide a certain treatment time using timers or by applying fixed circulation rates. However, because the characteristics of swine manure and the wastewater treatment efficiency of bioreactors change continuously, BCS cannot be operated effectively using conventional methods with fixed operating conditions. In contrast, the bioreactor operation strategy using ORP and pH (mV)-time profiles enables optimized BCS operation in response to fluctuations in loading rates and microbial activities. In this study, PLC was used to determine the real-time control point of the bioreactor by detecting NBP and NKP via ORP and pH (mV) monitoring. Consequently, the bioreactor could remove NH_4_-N completely while achieving effective real-time BCS control by optimizing the duration of the anoxic and aerobic phases. These results suggest that real-time control systems based on the time profiles of ORP and pH (mV) are effective for farm-scale BCS operations.

### 3.2. Quantitative Analysis for Bio-Liquor Circulation

**Figure 7:**
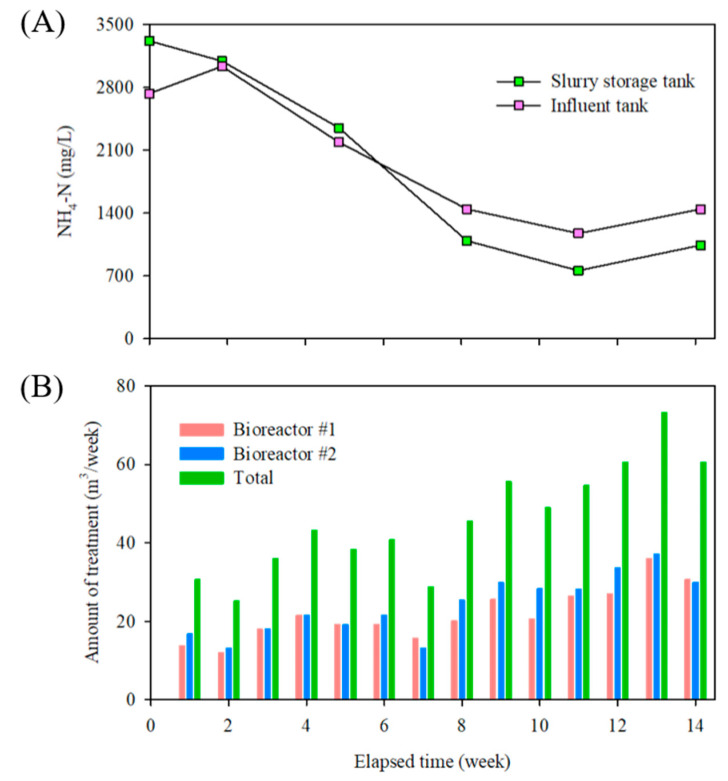
Change in the amount of bio-liquor circulated and the NH_4_-N concentration in the influent during the BCS operation under real-time controlled condition: (**A**) Trend of NH_4_-N concentration in the slurry storage tank and influent tank, (**B**) Changes in influent treatment amount.

Figure 7 shows that the amount of wastewater treated in the real-time controlled BCS increased over time. As the bio-liquor circulated continuously into the slurry pit, the concentration of NH_4_-N in the slurry storage and influent tanks decreased constantly (Figure 7A). Figure 7B illustrates the increasing pattern in the amount of wastewater treated. The total amount of wastewater treated in bioreactors 1 and 2 was 30.6 m^3^/week in the first week, which gradually increased over time to a maximum of 73.2 m^3^/week in the 13th week. The primary reason for this increase was the reduction in nitrogen and organic matter in the influent due to the circulation of the well-treated bio-liquor into the slurry pit. 

When the raw slurry manure was discharged into the slurry storage tank from the NCS-SP, where the bio-liquor did not circulate, the concentrations of nitrogen and organic matter in the influent might have increased rapidly. Therefore, the amount of wastewater treated in the bioreactor temporarily decreased at 2, 5, 7, 10, and 14 weeks (Figure 7B). 

In this study, the average amount of wastewater treated in the two bioreactors was 6.9 ± 2.2 m^3^/d, and the circulation rate based on the working volume of the slurry pit was 39.0 ± 12.3 L/m^3^/d (Table 7). In addition, as BCS-SP was full of swine manure at the beginning of the experiment, it took time to lower the concentrations of organic matter and nitrogen in the influent. Therefore, the circulation rate was low at the beginning of the experiment, but gradually increased as the circulation of the bio-liquor continued. In this study, since the circulation rate of the two bioreactors was as high as 10.5 ± 3.3 times the manure production in the BCS swine barn, it can be judged that a sufficient circulation rate can lower the concentrations of nitrogen and organic matter, which are the major causes of odor in slurry pits. Consequently, the NH_4_-N concentration in the slurry storage tank decreased continuously from the beginning of the bio-liquor circulation (Figure 7A), which played a crucial role in reducing the loading rate in the bioreactor. Consequently, an increase in the amount of bio-liquor circulation was observed from week 3 onwards compared to weeks 1–2 (Figure 7B).

### 3.3. Changes in Swine Manure Properties in Slurry Pit

The characteristics of the manure in NCS-SP and BCS-SP were compared (Table 8). The concentrations of TS, TVS, TSS, TVSS, NH_4_-N, and STOC in the BCS slurry pit were >70% lower than those in the NCS. Even TN, which had the lowest reduction efficiency, showed a significant decrease of 69.0% compared to that in the NCS. Notably, the NH_4_-N and STOC concentrations, which were less influenced by solids, showed remarkably high reduction efficiencies of 87.5% and 89.5%, respectively.

This result confirms that real-time-controlled BCS can effectively remove pollutants and odor sources from slurry pits. Wi et al. managed swine manure in the slurry pit by circulating aerobically treated manure (bio-liquor of their study) 10 times the amount of manure production [31,32]. They reported significant seasonal differences in the reduction rates of NH_4_-N within the slurry pit, with rates of 33.3% in summer and 78.9% in fall. In their study, the NH_4_-N concentration in the aerobically treated manure exhibited substantial differences between summer (1860 mg/L) and fall (216 mg/L). These variations influenced the improvement level of swine manure within the slurry pit. Remarkably, in the case of summer, even though the NH_4_-N concentration of swine manure in the slurry pit was 33.3% lower compared to the control group, NH_3_ emissions increased by 31%. These findings suggest that since the NH_3_ concentration which could be potentially emitted in the aerobically treated manure was high, at 322 mg/L (calculated using NH_4_-N concentration, pH, and the average summer temperature suggested by Wi et al.), it is possible that the circulation of aerobically treated manure itself increased NH_3_ emissions. These research results by Wi et al. clearly demonstrate why conventional BCS cannot be operated effectively, and explain why real-time controlled BCS in this study is important. 

To evaluate the effect of the real-time controlled BCS, the potential CH_4_ and NH_3_ emissions from NCS-SP and BCS-SP were estimated. The estimated potential CH_4_ emission from swine manure in the NCS-SP and BCS-SP was 13.2 ± 0.4 and 3.3 ± 0.1 kg CH_4_/m^3^ manure, respectively. These findings suggest that the implementation of real-time BCS can lead to a 75% reduction in CH_4_ generation from slurry pits. According to several studies, the circulation of bio-liquor into the slurry pit may inhibit the generation of CH_4_ from swine manure. During aerobic processes, aerobic microorganisms oxidize organic matter and produce stable humic substances (HS), which are poorly biodegradable during energy metabolism, competitively inhibiting the reduction of other terminal electron acceptors under methanogenic conditions [44,45,46,47,48]. The addition of HS reduces the production of CH_4_, and Khadem et al. found that HS treatment could effectively inhibit CH_4_ production by *Methanobacterium formicum*, *Methanobrevibacter arboriphilicus*, and *Methanosarcina barki* [49,50,51]. 

The potential amount of NH_3_ emissions from NCS-SP and BCS-SP was estimated to be 103.7 and 5.0 g NH_3_/m^3^ manure, respectively. These findings indicate that the NH_3_ emissions from the slurry pit can be reduced by 95.2% by implementing real-time BCS. NH_3_ is indeed an odorous gas emitted by swine farms and is widely recognized for its detrimental impact on the respiratory health of workers and the growth of livestock. Additionally, NH_3_ acts as a precursor in the formation of fine dust through photochemical reactions when released into the atmosphere [52,53]. Therefore, the application of real-time BCS could be a useful way to improve environmental conditions in swine farms by reducing the NH_3_ generated from manure and suppressing the production of fine dust precursors.

## 4. Conclusions

Improving environmental conditions and implementing proper manure management on swine farms are essential for enhancing swine productivity. In addition, reducing malodor and preventing environmental pollution are the top requirements for securing sustainability in the swine industry. BCS can be an effective way to improve the environment of swine farms by managing potential pollutants and odor sources contained in swine manure during storage. However, maintaining the optimal operating performance of a BCS under fixed-time operating conditions is challenging. This study shows that a real-time control strategy for bioreactors using ORP and pH (mV)–time profiles can effectively optimize BCS performance. The bioreactor, which was operated using a well-designed algorithm, provided 100% NH_4_-N removal and lowered over 70% of the organic matter and nitrogen in the slurry pit manure by optimizing the overall treatment process and circulation time in real time. The CH_4_ and NH_3_ potential emissions from the slurry pit also decreased by 75% and 95.2%, respectively. This novel real-time control strategy using ORP and pH (mV)–time profiles can always optimize the operational performance of the BCS under various conditions, such as changes in microbial activities and temperature and fluctuations in manure composition. The results of this study suggest novel system operation strategies for the effective and practical use of BCS. In the future, conducting studies on real-time controlled BCS on a larger scale will allow for further investigations into operational limitations, optimal process design scale, and more. Additionally, research on verifying odor and greenhouse gas emissions from swine facilities with real-time controlled BCS based on gas measurements should be conducted.

## Figures and Tables

**Table 1:** Specifications of our process in the experimental farm.

Item	Shape	Area (m^2^)	Depth (m)	Volume (m^3^)	
				Total	Working
NCS-SP	Rectangular Paralleled pipe	327.5	0.7	212.9	131.0
BCS-SP		440.3	0.7	286.2	176.0
Slurry storage tank		12.3	3.5	43.1	30.0
Influent tank		18.9	3.5	66.0	50.0
Bioreactor #1		16.7	3.5	58.5	40.0
Bioreactor #2		16.7	3.5	58.5	40.0
Distributer		2.5	1.2	3.0	2.0
Effluent tank	Cylindrical	34.2	3.0	100.0	80.0

**Table 2:** Operational conditions of the bio-liquor circulation system.

Parameters	Bioreactors 1 & 2	BCS-SP	NCS-SP
Working volume (m^3^)	40	176.0	131.0
Initial condition	Filled with bio-liquor	Full of slurry manure	
Manure production (L/d)	-	652.8	489.6
Working volume of slurry pit (m^3^/head)	-	2.28	2.24
Circulation rate (m^3^/cycle)	0.6	0.6	No circulation
Circulation rate based on working volume (L/m^3^/cycle)	15	3.4	No circulation
Aeration rate (m^3^/min/m^3^)	0.1	-	-

**Table 3:** Characteristics of mixed liquor used in the bioreactors.

Parameters (mg/L)	Mixed Liquor
TS ^1^	24,225.0
TVS ^2^	11,795.9
TSS ^3^	18,980.0
TVSS ^4^	9766.7
NH_4_-N ^5^	83.6
NO_X_-N ^6^	0.0
TKN ^7^	1066.1
TN ^8^	1066.1
STOC ^9^	1529.1

^1^ TS, total solids; ^2^ TVS, total volatile solids; ^3^ TSS, total suspended solids; ^4^ TVSS, total volatile suspended solids, ^5^ NH_4_-N, ammonium nitrogen; ^6^ NO_X_-N, nitrogen oxides; ^7^ TKN, total Kjeldahl nitrogen; ^8^ TN, total nitrogen; ^9^ STOC, soluble total organic carbon.

**Table 4:** Temperature range and feed composition.

Parameters		Breeding Room in NCS and BCS	
		FR 1 & 2	PR
Temperature (°C)	Max.	25	30
	Min.	20	25
Feed composition	Crude protein (%)	14–16	18–20
	Crude fat (%)	4–5	7–8
	Crude fiber (%)	3–5	2.5–5
	Crude ash (%)	6–7	4.5–5
	Calcium (%)	0.5–0.6	0.6–0.7
	Phosphorus (%)	0.35–0.4	0.6–0.65
	Lysine (%)	0.8–0.9	1.45–1.5
	Digestible energy (Mcal/kg)	2.4–2.46	2.45–2.5

**Table 6:** Obtained results of bioreactor operation and soluble nitrogen concentration in bio-liquor.

Parameters	NH_4_-N Loading Rate (g/m^3^·d)	F/M ^1^ (Ratio/d)	Circulated Bio-Liquor (mg/L)			
			MLSS ^2^	NH_4_-N	NO_X_-N	STOC
Bioreactor 1	157.1 ± 43.0	9.2 ± 3.8	25,185.0 ± 3933.0	0.0 ± 0.0	0.0 ± 0.0	1613.7 ± 75.2
Bioreactor 2	192.9 ± 46.3	11.0 ± 5.5	26,058.3 ± 5795.7	0.0 ± 0.0	0.0 ± 0.0	1640.8 ± 98.2

^1^ F/M, mg NH_4_-N/g MLVSS (mixed liquor volatile suspended solids); ^2^ MLSS, mixed liquor suspended solids.

**Table 7:** Results of quantitative analysis of bioreactor operation.

Parameters	Bioreactor 1	Bioreactor 2	Total
Amount of circulation (m^3^/d)	3.3 ± 1.1	3.6 ± 1.2	6.9 ± 2.2
Circulation rate based on slurry pit volume (L/m^3^/d)	18.6 ± 6.3	20.4 ± 6.6	39.0 ± 12.3
Circulation ratio based on manure production (bio-liquor/manure)	5.0 ± 1.7	5.5 ± 1.8	10.5 ± 3.3

**Table 8:** Comparison of the swine manure characteristics in slurry pit of the non-circulation system (NCS) and bio-liquor circulation system (BCS).

Parameters	NCS	BCS	Reduction Efficiency (%)
TS (mg/L)	172,037.5 ± 7548.4	50,421.3 ± 708.2	70.7
TVS (mg/L)	124,975.0 ± 3712.3	31,220.4 ± 858.4	75.0
TSS (mg/L)	151,597.7 ± 12,810.4	40,757.4 ± 1442.1	73.1
TVSS (mg/L)	112,716.7 ± 6976.8	28,102.8 ± 409.0	75.1
NH_4_-N (mg/L)	3061.1 ± 71.3	382.4 ± 190.6	87.5
TN (mg/L)	11,956.3 ± 2391.5	3710.8 ± 251.2	69.0
STOC (mg/L)	29,292.1 ± 884.2	3086.6 ± 432.5	89.5
pH	7.79 ± 0.03	7.20 ± 0.06	-
Potential amount of CH_4_ production (kg/m^3^)	13.2 ± 0.4	3.3 ± 0.1	75.0
Theoretical NH_3_ emissions (g/m^3^)	103.7 ± 4.1	5.0 ± 1.2	95.2

## Data Availability

The data presented in this study are available on request from the corresponding author. The data are not publicly available due to security concerns.
